# Does Diffusion Restriction Pattern on MRI Predict Stroke Etiology in a Cancer Patient?

**DOI:** 10.2174/1573405619666221230115119

**Published:** 2023-05-17

**Authors:** Mehmet Kolukisa, Bahar Aksay Koyuncu, Alişan Bayrakoglu, Talip Asil

**Affiliations:** 1 Department of Neurology, Bezmialem Vakıf University, Istanbul, Turkey;; 2 Department of Neurology, Memorial Hizmet Hospital, Istanbul, Turkey

**Keywords:** Ischemic stroke, etiology, cancer, diffusion MRI, DWI, HT

## Abstract

***Background*:** Stroke and cancer are two of the most common health problems. Moreover, stroke is more common in patients with cancer than in the normal population, due to coagulation problems. Knowing the etiology of stroke is important for determining treatment options. This study aimed to determine the relationship between ischemic lesion topographies using diffusion-weighted magnetic resonance imaging (MRI) and the etiology of stroke in patients with cancer.

***Patients and Methods*:** All patients with ischemic stroke in the Bezmialem Stroke Registry over a 4-year period were retrospectively analyzed in this study. Patients with acute ischemic stroke and additional diagnoses of solid and active malignancy (excluding hematologic malignancies) were included in the analysis. We investigated whether there was a relationship between the etiology of patients with cancer-related stroke according to the stroke etiologic classification and the diffusion restriction patterns on MRI.

***Results*:** In this registry, 32 of 1472 patients were diagnosed as having active cancer. Fourteen patients were evaluated as having definite cardioembolism, eight patients as probable cardioembolism, and four patients had inadequate examinations. Only one patient was classified as having an atherothrombotic stroke. Isolated acute infarction was seen in 15 of 32 patients. In patients with multiple acute infarct areas (n=17), acute lesions characterized by micro embolisms in a single vessel area were detected in four patients, and acute lesions characterized by bilateral (anterior and/or posterior system) micro embolisms in more than one vessel area in 13 patients.

***Conclusion*:** The most common etiology of stroke in patients with cancer was found to be embolic/cardioembolic. This is important for the treatment plans for ischemic stroke in patients with cancer.

## 1 INTRODUCTION

Both ischemic stroke and cancer are frequent diseases that cause mortality and morbidity [1, 2]. The coexistence of these two diseases is not rare. Approximately 15% of patients with cancer have the cerebrovascular disease, whereas approximately 20% of patients with a stroke of undetermined cause (cryptogenic stroke) may have an occult malignancy at the time of stroke [3].

It is difficult to diagnose cancer-related stroke because cancer is underestimated in these patients due to the lack of specific diagnostic markers. It is also not easy to detect the exact cause of stroke in cancer patients because they mostly share similar risk factors like age or smoking. These cancer patients may have a stroke from not only Troussaud syndrome [4] but also well-established causes like a small-vessel disease. Atrial fibrillation is also known to have a relationship with occult cancer [5, 6].

Cryptogenic stroke was the most frequent subtype reported by the studies, investigating TOAST subtypes among patients with ischemic stroke and comitant malignancy. [6-8]. On the Contrary, Cestari *et al*. [9] reported embolic ischemic stroke to be more common than non-embolic in ischemic stroke patients with underlying cancer. It has been discussed for a long time that the TOAST classification is insufficient to clearly define the underlying cause of stroke etiology [7], and other classifications have been proposed to help the patient reach more targeted therapy by classifying the stroke etiology more effectively [10]. The ASCOD [11] classification covers all the causes underlying the stroke etiology and classifies them according to their severity. While making this classification, the location of the lesions seen in the Diffusion-weighted imaging (DWI) MRI, leukoareosis, and the presence of microhemorrhages are taken into consideration. The etiology of stroke can be detected in more patients in ASCOD and similar classifications compared to the TOAST classification [12].

DWI is a commonly performed magnetic resonance imaging (MRI) sequence for the evaluation of acute ischemic stroke and is sensitive to the detection of small and early infarction [13]. Many studies have attempted to unravel stroke etiopathogenesis through ischemic lesion topography using DWI [14, 15]. It has been reported that multiple lesions in one vascular area are related to large artery atherosclerosis [14]. Perforating infarcts, in addition to pial or border zone infarcts, were distinctive patterns for intracranial atherosclerosis. DWI can also enable the detection of small lacunar infarcts [7, 8]. Recent studies showed that lesion patterns detected using DWI MRI might help to understand the underlying stroke etiopathogenesis in patients with cancer [14-17].

Our study aimed to determine the relationship between ischemic lesion topographies on DWI MRI and the etiology of stroke in patients with cancer so we think that determining the etiology of stroke is the first step for secondary prevention.

## 2 PATIENTS AND METHODS

All patients with ischemic stroke who were admitted to the Neurology Department of the Bezmialem Vakif University between January 2018 and December 2021 were retrospectively analyzed in this study. Patients with acute ischemic stroke and additionally diagnosed with cancer who had solid and active malignancies (excluding hematologic malignancies) were included in the analysis. Active cancer was defined as confirmed malignancy in patients with metastatic cancer treated or within the last 6 months prior to the stroke. Diagnosis of cancer was confirmed through given medical records or, in the case of newly diagnosed or recurrent cancer, with histologic evidence and oncologist expertise.

The following criteria were described as vascular risk factors: history of hypertension (HT) or observed arterial blood pressure (140/90 mm Hg); a history of diabetes mellitus (DM) or fasting glucose exceeding 126 mg/dL, except those measured during the acute phase; a history of positive hyperlipidemia (HL) or fasting total cholesterol (200 mg/dL, low-density lipoprotein (LDL) (100 mg/dL) and triglyceride (TG) (180 mg/dL)). Data for the presence of coronary artery disease (CAD), coronary artery bypass grafting (CABG), smoking, and previous history of stroke were recorded from patients’ medical records. The diagnostic examination was analyzed according to data collected within the first 3-month period after the index stroke. The diagnostic workup included cranial and vascular imaging techniques, 12-lead electrocardiography (ECG), transthoracic and transesophageal echocardiography, when necessary (TTE and TEE, respectively), 24-h Holter electrocardiography (ECG) monitoring. Coagulation parameters (protein C, protein S, antithrombin III, prothrombin II, and factor V Leiden) and vasculitis markers (lupus anticoagulants, anticardiolipin antibody, antinuclear antibodies, anti-DNA, antineutrophil cytoplasm antibodies) were also investigated if a patient was younger than 50 years old (labeled young stroke), and conventional angiography, if needed.

Considering all variable data, a stroke physician assigned all patients to an etiologic subtype using the ASCOD [11] and TOAST [10] classification systems. With the ASCOD classification, patients were reviewed for the presence of each of the following etiologic mechanisms: atherosclerosis (A), small vessel disease (S), cardioembolic (C), and other causes (O). Etiologic mechanisms are classified as grade 1, 2, 3, 0, or 9 per the following: grade 1 if the mechanism is a potential cause of the index stroke, grade 2 if the causality is uncertain, grade 3 if the disease is present but unlikely to be the cause of the index stroke, grade 0 if the disease is absent, and grade 9 if the diagnostic examination is insufficient.

### 2.1 MRI Assessment and Analysis

The MRI investigations were performed on 1.5-Tesla MRI scanners. The scanning protocol included axial DWI, obtained with a 5-mm slice thickness. The acute lesion pattern on DWI was characterized according to number and localization regarding the affected vascular territory: (a) Single acute lesion; (b) Multiple acute lesions in one vascular territory with (micro-) embolic scattering of infarction; (c) Multiple acute lesions in >1 vascular territory (bihemispheric anterior circulation lesions0 without (micro-) embolic scattering of infarction. (d) Multiple acute lesions in >1 vascular territory (bihemispheric anterior circulation lesions) with (micro-) embolic scattering of infarction; (e) Multiple acute lesions in >1 vascular territory (anterior and posterior circulation lesions) with (micro-) embolic scattering of infarction [8]. In addition, subacute (DWI hyper- and apparent diffusion coefficient iso- or hyperintense) and chronic ischemic lesions were assessed. Images were reviewed independently by two stroke neurologists (K.M. and K.B.A.0. In cases of discrepancy, the final pattern classification was reached by mutual agreement of the readers.

We investigated whether there was a relationship between the etiology of patients with cancer-related stroke according to the stroke etiologic classification and the diffusion restriction patterns detected in DWI MRI.

### 2.2 Statistical Analysis

The SPSS 16 for Windows statistical software package was used for statistical analysis. All parameters are presented as mean +/- standard deviation (SD). Data comparisons were performed using the Chi-square test and Student’s t-test. The significance of the results was assessed using P values; P-values of <0.05 were considered statistically significant.

## 3 RESULTS

Between 2018 and 2021, 1472 patients were registered consecutively in the Bezmialem Vakif University Stroke Registry. In this registry, 32 patients were diagnosed as having active cancer. The mean age of the patients was 65 (46-89; median: 65) years and 59.4% were male. The distribution of vascular risk factors is shown in Table **1**. Gastrointestinal system malignancies were the most common in these patients.

Ischemic stroke etiologies of these patients were evaluated according to the ASCOD and TOAST classifications, separately (Table **2**). According to the ASCOD classification, 14 patients were evaluated as having definite cardioembolism (C1), eight patients as probable cardioembolism (C2), and four patients had inadequate examinations. Only one patient was classified as having an atherothrombotic stroke.

When the etiology of stroke was classified according to the TOAST classification, one patient was evaluated as having an atherosclerotic stroke, 14 patients had definite cardioembolic strokes, nine patients had strokes of unknown causes, and eight patients had an undetermined stroke due to insufficient investigations. No small vessel disease, other rare causes, or dissection was detected.

The lesions detected in diffusion MRI were grouped according to the classification given in Tables **3a** and **3b**. Isolated acute infarction was seen in 15 of 32 patients. In patients with multiple acute infarct areas (n=17), acute lesions characterized by micro embolisms in a single vessel area were detected in four patients, and 13 patients had acute lesions characterized by bilateral (anterior and/or posterior system) micro embolisms in more than one vessel area. We detected no typical lacunary infarctions. Diffusion MRI images of five different patients are presented in Figs. (**1a**-**e**).

When the patients were divided into two groups according to the presence/absence of isolated acute infarct, no statistically significant difference was observed in terms of demographic characteristics and vascular risk factors of these patients. Hypertension frequency was significantly higher only in the group with isolated infarcts (73.3% *vs*. 35.5%; p=0.042). When the etiologic classifications of stroke were examined, no statistically significant difference was found between the two groups, regardless of whether it was the ASCOD or TOAST classification. Nine out of 15 patients with isolated acute infarction had atrial fibrillation (AF). When the patients with AF (n=14) and large vessel occlusion (n=1) were excluded, it was observed that 12 (70.6%) of the remaining 17 patients who had no definite etiology, had multiple acute lesions. Although it did not reach statistical significance due to the small number of patients, it was observed that multiple lesions were more common in patients with a history of active malignancy, even in those without AF.

## 4 DISCUSSION

Ischemic stroke and cancer are epidemiologically and mechanistically linked diseases that will likely increase in co-prevalence as survival from cancer improves. Active cancer is a well-known risk factor for ischemic stroke. Among patients with ischemic stroke, approximately 10% of them have known cancer. Additionally, 3% have hidden cancer [18]. Many studies have shown an increased risk of stroke and other arterial thromboembolic events in patients with cancer compared with matched controls [19-21]. This risk is particularly high in the first 6 months after cancer diagnosis and distant metastases [22]. In our study, 32 of 1472 (2.1%) patients with ischemic stroke had active or metastatic cancer that was diagnosed/treated within the last 6 months. Also, the risk of stroke varies with the type of cancer. This risk is highest in cancers that are mostly associated with an increased risk of venous thromboembolism, particularly lung and pancreatic cancer [21, 22]. In our study, the lung and gastrointestinal systems were the most frequent cancer types.

In the past, stroke subtypes were not statistically different between patients with ischemic stroke with and without cancer [8, 23, 24]. In recent studies, it has been found that embolic strokes are more common in patients with cancer than in other etiologies [25]. In previous studies, the etiology of stroke subtypes was classified according to the TOAST criteria. Cestari *et al.* [9] found that thrombotic strokes [54%], including cardio-aortic embolic strokes (15%), were more common than non-thrombotic (46%) strokes, including those of undetermined and atherosclerotic ischemic strokes. Kim *et al.* [26] found that the etiology of stroke subtypes was large artery atherosclerosis (41%), small artery occlusion (28.8%), cardio-aortic embolism (17.3%), etiology of uncertain (8.3%), and other stroke subtypes. In our study, it was shown that cardioembolic strokes were significantly more common than other etiologies. Although large artery atherothrombosis was detected in only one patient out of 32 patients, no small vessel disease was detected in any patients. This distribution did not change whether we used the TOAST or ASCOD classifications. However, the number of embolic strokes increased in the ASCOD classification, whereas fewer patients remained of unknown cause. In the ASCOD classification, both the embolic source investigation and DWI lesion distribution patterns are taken into consideration. The reason why more embolic strokes were detected in the ASCOD classification than in the TOAST classification is probably due to our more active use of neuroimaging in the ASCOD classification.

Schwarzbach *et al*. showed that [5] DWI lesions in multiple vascular supply territories strongly dominated the phenotype of cancer-related stroke, being observed in 84% of all patients included in their analysis. Additionally, (micro-) embolic scattering is a frequent feature of ischemic infarction in this special cohort of patients. In our study, multiple embolisms, which can be thought to be of embolic origin, were detected on DWI MRI in 17 patients (53%), 88.2% (n=15/17) of these were with micro-scattering. Apart from these, nine of 15 patients with single DWI lesions were also shown to have AF. When it is difficult to determine the etiology in patients with cancer and stroke, cardiac examinations need to be performed insistently even in patients with single lesions on DWI.

Intravascular coagulopathy is the most common stroke mechanism proposed in cancer patients. In patients with cancer-related coagulation disorders, intravascular thrombus may occur despite the absence of a large nidus for thrombus formation/dissemination, such as a heart valve or embolic sources in the deep vein. As a result, stroke caused by intravascular coagulation disorder typically presents as multiple small infarcts at multiple sites [27].

DWI MRI is a much more sensitive tool than cranial CT to evaluate embolic brain infarctions, especially small single or multiple infarcts in the cortical or subcortical areas, in patients with acute ischemic stroke. Cardiogenic, paradoxical, or artery-to-artery emboli are common sources of embolic ischemic stroke. These embolisms can be detected by transesophageal echocardiography (TEE). However, in 16% of embolic stroke patients, embolic sources cannot be determined, so early imaging with DWI MRI is very helpful to diagnose embolic stroke [28].

This study also had some limitations. The main limitations are that it is a single-center, retrospective study with a limited number of patients.

Our study showed that when all tests for the etiology of stroke were performed in detail and evaluated together with DWI MRI, the most common etiology of stroke in patients with cancer was found to be embolic/cardioembolic. Anticoagulant therapy has not yet proven its efficacy in Admitted patients with ESUS [29, 30]. In cancer patients, anticoagulant therapy for 6 months is recommended in the presence of venous TE, but the benefit of longer use is not fully known [31]. Information on the effectiveness/duration of anticoagulant therapy in cancer patients with ischemic stroke is still limited [25]. For this reason, there is a need to investigate the efficacy of anticoagulant therapy in cancer patients with ischemic stroke, especially in patients who do not have clear AF but are thought to have a possible cardioembolic nature.

## CONCLUSION

In this study, we showed that cardioembolic and embolic strokes have an important role in the etiology of stroke in patients with cancer. The use of MRI in patients with stroke and cancer will be helpful in determining stroke etiology. Conducting multicenter studies on this subject will help in the management of secondary prevention.

## Figures and Tables

**Fig. (1):**
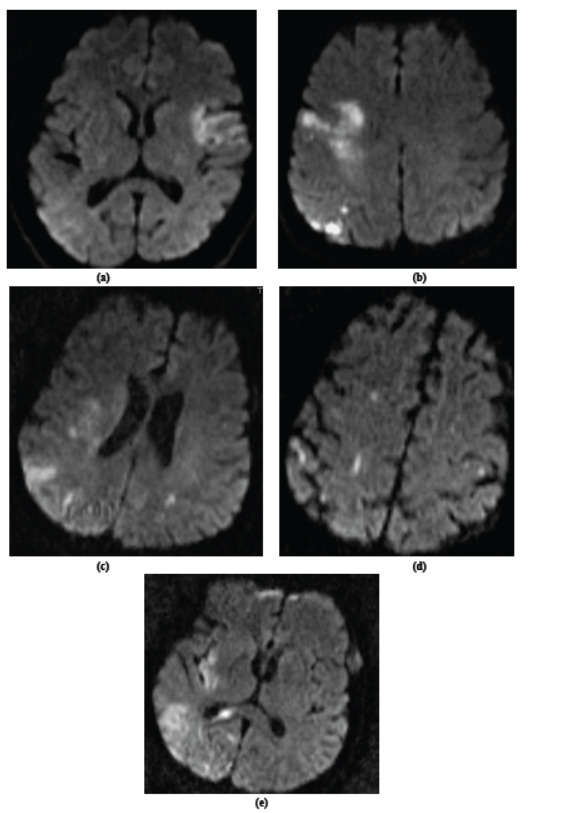
(**a**) Single acute lesion. (**b**) Multiple acute lesions in one vascular territory with (micro-) embolic scattering of infarction; (**c**) Multiple acute lesions in >1 vascular territory (bihemispheric anterior circulation lesions) without (micro-) embolic scattering of infarction. (**d**) Multiple acute lesions in >1 vascular territory (bihemispheric anterior circulation lesions) with (micro-) embolic scattering of infarction; (**e**) Multiple acute lesions in >1 vascular territory (anterior and posterior circulation lesions) with (micro-) embolic scattering of infarction [8].

**Table 1:** Distribution of demographic and risk factors in patients with single and multiple lesions on diffusion-weighted magnetic resonance imaging.

-	**All Patients** **(n=32)**	**Single Lesions** **(n=15)**	**Multiple Lesions (n=17)**	**p**
**Age mean** **(minimum-maximum; median)**	65 (46-89; 65)	65 (50-75; 67)	66 (46-89; 65)	0.900
**Male % (n)**	59.4 (19/32)	53.3 (8/15)	64.7 (11/17)	0.720
**DM % (n)**	28.1 (9/32)	33.3 (5/15)	23.5 (7/17)	0.699
**HT % (n)**	53.1 (17/32)	73.3 (11/15)	35.3 (6/17)	**0.042**
**HL % (n)**	37.5 (12/32)	46.7 (7/15)	29.4 (5/17)	0.467
**CAD % (n)**	21.9 (7/32)	26.7 (4/15)	18.8 (3/17)	0.685
**Stroke history % (n)**	15.6 (5/32)	20 (3/15)	11.8 (2/17)	0.645
**AF % (n)**	47.8 (11/23)	7.1 (8/11)	33.3 (3/9)	0.400
**Antithrombotic use % (n)**	43.3 (13/30)	46.2 (6/13)	41.2 (7/17)	0.999
**GIS CA % (n)**	34.4 (11/32)	33.3 (5/15)	35.3 (6/17)	0.999
**Pulmonary CA % (n)**	21.9 (7/32)	13.3 (2/15)	29.4 (5/17)	0.402
**Early recurrent stroke % (n)**	31 (9/29)	30.8 (4/13)	31.3 (5/16)	0.999

**Abbreviations:** DM, Diabetes Mellitus; HT, Hypertension; HL, Hyperlipidemia; CAD, Coronary Artery Disease; AF, Atrial Fibrillation; GIS, Gastrointestinal System; CA, Cancer

**Table 2:** ASCOD and TOAST classifications in patients with cancer and ischemic stroke.

**ASCOD**	**All Patients** **(n=32)**	**Single Lesions** **(n=15)**	**Multiple Lesions (n=17)**	**p**
Atherosclerosis % (n)	3.1 (1/32)	6.7 (1/15)	0	-
Cardioembolic % (n)				
C0 (no cardiac source)	15.6 (5/32)	20 (3/15)	11.8 (2/17)	0.645
C1 (Definite - AF present) %(n)	43.8 (14/32)	60 (9/15)	29.4 (5/17)	0.153
C2 (Probable- with multiple infarct) %(n)	25 (8/32)	6.7 (1/15)	41.2 (7/17)	0.041
C9 (only ECG) %(n)	12.5 (4/32)	6.7 (1/15)	17.6 (3/17)	0.603
Small VD %(n)	0	0	0	
Other Causes%(n)	0	0	0	
Dissection%(n)	0	0	0	
TOAST	All patients (n=32)	Single lesions (n=15)	Multiple lesions (n=17)	p
LVD %(n)	3.1 (1/32)	6.7 (1/15)	0	0.469
SVD %(n)	0	0	0	-
CE %(n)	43.8 (14/32)	60 (9/15)	29.4 (5/17)	0.153
Undetermined (negative+incomplete) %(n)	53.1 (17/32)	33.3 (5/15)	70.6 (12/17)	0.035*
UND-negative evaluation %(n)	28.1 (9/32)	26.7 (4/15)	29.2 (5/17)	0.999

**Abbreviations:** LVD, Large vessel disease; SVD, Small vessel disease; CE, Cardioembolic; UND, Undetermined

**Table 3a:** Lesion patterns on diffusion-weighted magnetic resonance according to the ASCOD classification.

**DWI/ASCOD**	**A**	**C0**	**C1**	**C2**	**C9**	**Total**
A	1	3	9	1	1	15
B	0	2	0	1	1	4
C	0	0	1	1	0	2
D	0	0	2	4	1	7
E	0	0	2	1	1	4
Total	1	5	14	8	4	32

**Table 3b:** Lesion patterns on diffusion-weighted magnetic resonance according to the TOAST classification.

**DWI/TOAST**	**Cardioembolic**	**Atherothrombotic**	**Cryptogenic**	**Incomplete Evaluation**	**Total**
A	9	1	4	1	15
B	0	0	3	1	4
C	1	0	0	1	2
D	2	0	1	4	7
E	2	0	1	1	4
Total	14	1	9	8	32

**Note:** *(A) Single acute lesion; (B) Multiple acute lesions in one vascular territory with (micro-) embolic scattering of infarction; (C) Multiple acute lesions in >1 vascular territory (bihemispheric anterior circulation lesions) without (micro-) embolic scattering of infarction. (D) Multiple acute lesions in >1 vascular territory (bihemispheric anterior circulation lesions) with (micro-) embolic scattering of infarction; (E) Multiple acute lesions in >1 vascular territory (anterior and posterior circulation lesions) with (micro-) embolic scattering of infarction.

## Data Availability

Not applicable.

## LIST OF ABBREVIATIONS

CABG: Coronary Artery Bypass Grafting
CAD: Coronary Artery Disease
DM: Diabetes Mellitus
DWI: Diffusion-Weighted Imaging
HL: Hyperlipidemia
HT: History of Hypertension
LDL: Low-Density Lipoprotein
MRI: Magnetic Resonance Imaging
TG: Triglyceride

## STANDARDS OF REPORTING

STROBE guidelines were followed.
